# The Architecture of Monospecific Microalgae Biofilms

**DOI:** 10.3390/microorganisms7090352

**Published:** 2019-09-13

**Authors:** Andrea Fanesi, Armelle Paule, Olivier Bernard, Romain Briandet, Filipa Lopes

**Affiliations:** 1 Laboratoire Génie des Procédés et Matériaux (LGPM), CentraleSupélec, Université Paris-Saclay, 91190 Gif-sur-Yvette, France; 2 Université Côte d’Azur, Inria, BIOCORE, BP 93, 06902 Sophia Antipolis Cedex, France; 3 Micalis Institute, INRA, AgroParisTech, Université Paris-Saclay, 78350 Jouy-en-Josas, France

**Keywords:** biofilm, microalgae, architecture, confocal laser scanning microscopy, FTIR spectroscopy

## Abstract

Microalgae biofilms have been proposed as an alternative to suspended cultures in commercial and biotechnological fields. However, little is known about their architecture that may strongly impact biofilm behavior, bioprocess stability, and productivity. In order to unravel the architecture of microalgae biofilms, four species of commercial interest were cultivated in microplates and characterized using a combination of confocal laser scanning microscopy and FTIR spectroscopy. In all the species, the biofilm biovolume and thickness increased over time and reached a plateau after seven days; however, the final biomass reached was very different. The roughness decreased during maturation, reflecting cell division and voids filling. The extracellular polymeric substances content of the matrix remained constant in some species, and increased over time in some others. Vertical profiles showed that young biofilms presented a maximum cell density at 20 μm above the substratum co-localized with matrix components. In mature biofilms, the maximum density of cells moved at a greater distance from the substratum (30–40 μm), whereas the maximum coverage of matrix components remained in a deeper layer. Carbohydrates and lipids were the main macromolecules changing during biofilm maturation. Our results revealed that the architecture of microalgae biofilms is species-specific. However, time similarly affects the structural and biochemical parameters.

## 1. Introduction

In the last decades, microalgae have been recognized as a valuable source of bioproducts such as pigments, anti-oxidants, and food supplements, and they have gained popularity in a wide range of commercial activities. In conventional photobioreactors (PBRs), microalgae present low biomass concentrations (1–3 gL^−1^; [1,2]), and 12 to 2000 L of liquid medium are required for the production of 1 Kg of microalgae dry mass [3]. This high water fraction requires energetic expenses for culture agitation (up to 385.71 MJ·Kg^−1^; [2]) and for biomass harvesting, dewatering, and drying (up to 82 MJ·Kg^−1^; [2]). Therefore, biomass production in typical PBRs is constrained by high energy and operating costs [4].

Biofilm-based cultivation systems are promising technologies overcoming the drawbacks of conventional PBRs. Such systems can reach high productivities (up to 35 g DW m^−2^ day^−1^) and biomass concentration (up to 96 gL^−1^; [2]). Furthermore, harvesting is simply carried out by scraping the attached biomass with minimal energy demand [3]. Finally, there is an increasing interest from the industrial sector about the great variety of molecules excreted by microalgae when developing biofilms [5]. Therefore, biofilm-based systems seem to address most of the challenges of suspended cultures.

In a biofilm, microbial cells are associated with a surface and enclosed in a matrix, which is mainly composed of water, polysaccharides, proteins, and nucleic acids [6]. The spatial arrangement of microorganisms and matrix components define the size and quantity of voids and channels, altering in turn the transport of nutrients and gases [7]. Therefore, the biofilm architecture induces marked gradients of nutrients, gases, and light along depth, inducing the cells to acclimate or displace in order to maintain an optimal growth [8,9]. Therefore, structural data are of major importance to better understand the complex behavior of biofilms (i.e., development and activity) and to improve the productivity of biofilm-based technologies.

Structural changes in bacteria biofilms have been well characterized experimentally under several growth conditions, and it has been shown that the architecture is strongly species and strain-dependent [10,11,12,13]. From a compositional point of view, it has been shown that changes of structural parameters in bacterial biofilms are strongly correlated to the biochemical composition of exopolymers [14,15,16,17]. Phototrophic biofilms, especially microalgae biofilms, have been far less studied [8,18,19,20,21,22]. In particular, little is known about the link between architecture and extracellular polymeric substances production for different species. In addition, the role played by the matrix in biofilm development has been only addressed in studies regarding mixed communities (i.e., bacteria, microalgae, etc.) [8,23,24], even though microalgae are known to excrete exopolymers with specific carbohydrates:proteins:lipids:nucleic acids ratios [25,26].

Therefore, in this work, we aimed at better understanding how various microalgae monospecific biofilms differ in their architecture and composition depending on the species. Four biofilm-forming microalgae species of actual or potential biotechnological interest were selected, including two green algae, a red algae, and a diatom. Biofilm structural dynamics and macromolecular composition were characterized by a combination of non-destructive techniques including confocal laser scanning microscopy (CLSM, which allows characterizing several structural parameters such as biovolume, thickness, roughness, and diffusion distance) and vibrational spectroscopy (ATR-FTIR spectroscopy).

## 2. Materials and Methods

### 2.1. Microalgae Strains and Planktonic Culture Maintenance

*Chlorella vulgaris* SAG 211–11b (Göttingen, Germany) was grown in 3N-Bristol [27], and the marine strain *Chlorella autotrophica* CCMP 243 (Bigelow, ME, USA) was grown in artificial seawater [28], whereas the diatom *Cylidrotheca closterium* AC170 (Caen, France) and *Porphyridium purpureum* SAG 1380–1e (Göttingen, Germany) were cultivated in filtered natural seawater. The marine media were supplemented with Walne’s medium [29]; 1 mL·L^−1^). All biofilms were inoculated from suspended stock cultures grown in a PSI MC1000 multicultivator (Photon systems instruments, Drásov, Czech Republic) in borosilicate tubes filled with 70 mL of growth medium. The cultures were bubbled and maintained semi-continuously at 25 °C under a continuous photon flux density of 80 μmol photons m^−2^ s^−1^. Cells from the stock cultures were harvested during the exponential phase (cell density of 2–3 × 10^6^ cell mL^−1^).

### 2.2. Biofilms Cultivation: Inoculum, Initial Adhesion, and Growth

Biofilms were grown in polystyrene μClear ^®^ 96-well microplates (Grenier Bio-one, Les Ulis, France). The inoculum was prepared by diluting a volume of suspended cells (see above) to a final concentration of 1 × 10^6^ cell mL^−1^ (1 × 10^5^ cell mL^−1^ for *C. closterium*) and by transferring 250 μL of such suspension in the wells. This cell concentration corresponded to a similar starting biovolume for all the species (~5 μm^3^ μm^−2^). Then, the cells were left for 24 h to adhere to the surface of the wells, and subsequently, 200 μL of the medium were removed in order to eliminate any unattached cell. After the first 24 h, 80 μL of medium were removed and replaced with new medium every two days to compensate for evaporation and to buffer nutrient and CO_2_ limitations. A continuous photon flux density of 100 μmol photons m^−2^ s^−1^ was used. Light (PAR 400–700 nm) was homogeneously provided by two sets of light emitting diodes (Alpheus LED, Montgeron, France). Biofilms growth was monitored for 11 days, and each day, the biofilms were scanned using CLSM to detect cells signal. At day 2, 7, and 11, the biofilms were also stained to characterize the matrix, and samples were harvested for ATR-FTIR spectroscopy.

### 2.3. Confocal Laser Scanning Microscopy (CLSM): Cells and Matrix Characterization

Images (512 × 512 pixels) were acquired using an inverted Zeiss LSM700 confocal microscope (Carl Zeiss microscopy GmbH, Jena, Germany) controlled using the Zen 10.0 software black edition (Carl Zeiss microscopy GmbH, Jena, Germany). All biofilms were scanned with a LD Plan-Neofluar 20x/0.4 Korr M27 objective with a 0.4 N.A. (numerical aperture). Each image was 638 × 638 μm in size with a z-step of 3.94 μm. The settings of the confocal microscope are reported in Table S1. After preliminary trials, a low magnification lens was preferred to a higher one (e.g., 63×), because it allowed scanning wider biofilm areas (which is essential to properly capture microalgae cell patterns) in a relatively short time and acquiring z-stacks over the whole biofilm depth. However, we have to point out that this choice comes with side effects, such as a greater point spread function and lower image resolution.

Two laser lines were used to respectively detect microalgae and the matrix surrounding them. Microalgae cells were observed by detecting chlorophyll *a* autofluorescence. Lectins and dextran (3 kDa), both labeled with fluorescein (FITC), were added at days 2, 7, and 11 to detect glycoconjugates and to visualize the matrix non-specifically, respectively [30,31,32,33]. The pool of extracellular glycoconjugates (i.e., exopolysaccharides, glycoproteins, etc.) specifically detected by the lectins will be identified here as EPS (extracellular polymeric substances). Dextran, on the other hand, is supposed to diffuse into the channels and voids, and get finally non-specifically trapped into the matrix [30,31,32,33]. A fresh cocktail of 20 lectins (Kits I, II, and III, Vector Laboratories, Peterborough, UK) or dextran were supplied at a final concentration of 13 μg mL^−1^. Then, the microplates were incubated in the dark for 30 min. Afterwards, the excess of dyes present in the wells was removed by carefully removing 140 μL of the medium and by adding another 140 μL of fresh medium (specific for each species) in each well. This process was repeated one time for the dextran and two times for the lectins. We have to point out that since a mixture of lectins was used to detect the matrix, it is possible that the interaction among the single lectins and the competition for similar targets may have occurred.

Chlorophyll *a* was excited with the 639-nm line of a 5-mW solid-state diode laser, and the emission of chlorophyll *a* autofluorescence was observed using the long pass (LP) filter, which was 615 nm. Lectins and dextran were excited with the 488-nm laser line of a 10-mW solid-state diode laser, and their fluorescence was detected using the band pass (BP) filter, which was 490–530 nm. Unlabeled organisms and wells filled with growth media but not inoculated with microalgae were used as a staining control. Each well was scanned on at least three random positions (three z-stacks), resulting in a total surface area of at least 1.2 mm^2^.

### 2.4. Image Analysis

The plug-in COMSTAT 2.1 (Technical University of Denmark; [11]) running in ImageJ 1.48v [34] was used to extract from the images the quantitative parameters typically used to characterize biofilm structures. The complete list of parameters is reported in Table S2. Images binarization was automatically computed in the plug-in by selecting a threshold value using the Otsu algorithm [35], and the function “connected volume filtering” was unchecked.

Since the autofluorescence of the cells comes from the chlorophyll within the chloroplasts, we have to point out that the structural parameters calculated from the images reflect such organelles rather than the whole cell, even though the overlapping of fluorescence and transmission images revealed good matching of the two acquisition modes (data not shown). However, to be consistent with the terminology present in most of the literature, we considered that the autofluorescence of the chlorophyll quantifies the cells.

### 2.5. ATR-FTIR Spectroscopy

At days 2, 7, and 11, the biofilms were scraped from four wells for each species. The samples were centrifuged at 8000× *g* for 5 min, and the supernatant was removed. After that, 1 mL of distilled water in the case of *C. vulgaris* and 1 mL of a solution of NaCl (35 gL^−1^) for the marine species were used to wash the biofilm suspensions from salts, which would otherwise interfere with the cell and matrix spectral signature. Afterward, the pellet was re-suspended in 5–10 μL of distilled water or NaCl, 1.5 μL were transferred on a 45° ZeSe flat crystal of an ATR-FTIR PerkinElmer Spectrum-two spectrometer (PerkinElmer, Waltham, MA, USA), and the sample was dried at room temperature for 20 min. Spectra were acquired in the range of 4000 to 400 cm^−1^ using 32 accumulations at a spectral resolution of 4 cm^−1^. Before each measurement, the empty crystal was measured using the same instrumental setting and used as a blank.

Spectra were baselined using the rubber band algorithm, and the ratios between the main macromolecular pool (proteins, lipids, and carbohydrates) were calculated as the ratios between the maximum absorption values for the spectral ranges corresponding to each macromolecular pool: proteins (Amide I; 1700–1630 cm^−1^), lipids (C=O; 1750–1700 cm^−1^), and carbohydrates (C–O–C, C–C and Si–O–Si in diatoms; 1200–950 cm^−1^). Since no separation between cells and matrix components was performed, the spectra reflected both the physiological changes occurring in the cells and those related to EPS.

### 2.6. Statistics

Statistics was performed using GraphPad prism 5.0 (San Diego, CA, USA) and R [36]. One-way and two-way ANOVA were used to test the statistical significance of mean differences among different species and over time. The level of significance was always set at 5%. The logistic function [37,38] was fit to the biovolume versus time curves, and the maximal cell biovolume (i.e., the biovolume at the plateau) and the specific growth rate (*μ*) were obtained in order to make quantitative comparisons between the species.

Correlation matrices, using Pearson’s coefficient, were computed using the package “corrplot” [39] present in R to investigate the relationship among structural parameters (obtained from CLSM) and biofilms macromolecular composition (FTIR ratios).

All results are reported as mean and standard deviations of several independent biological replicates. Biofilm dynamics were repeated on at least four independent microplates. In each microplate, for each species and for each time point, at least three separate wells were analyzed by CLSM. In each well, at least three randomly chosen biofilm areas were scanned.

## 3. Results

### 3.1. Biofilm Development over Time: Structural Characteristics

**Figure 1:**
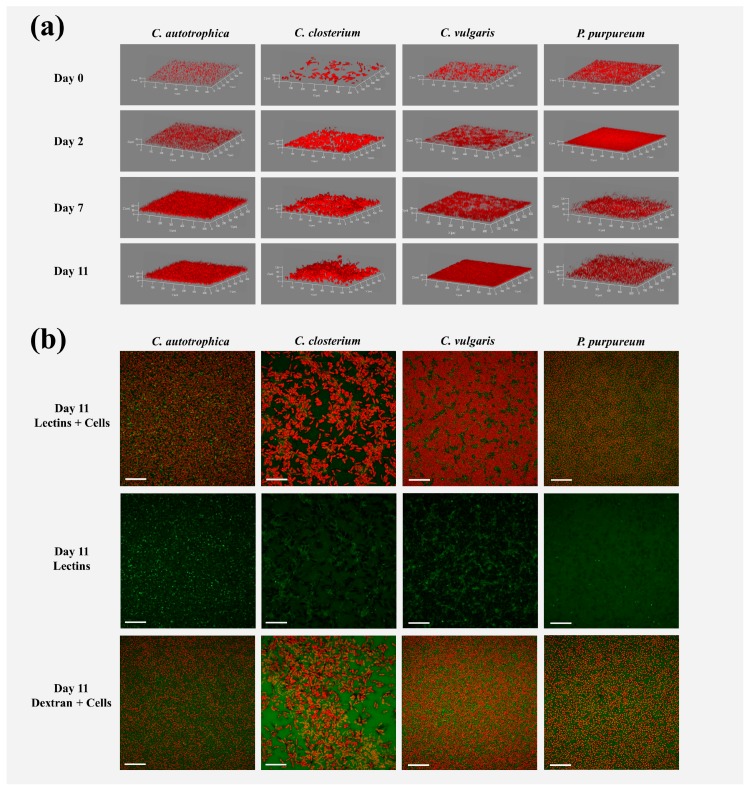
Representative three-dimensional reconstructions of the microalgae biofilms (**a**) and maximum intensity projection (**b**) of cells (red signal), lectins (green signal), and dextran (green signal) signals of the biofilms at day 11. In panel (**a**), day 0 represents the inoculum, while days 2, 7, and 11 are the days at which the biofilms were stained and analyzed by ATR-FTIR spectroscopy. The brightness of the images was adjusted for better visualization. The image’s size in (**a**,**b**) is 638 × 638 μm. The XY ticks interval in (**a**) is 100 μm, and the scale bar in (**b**) is 100 μm.

**Figure 2:**
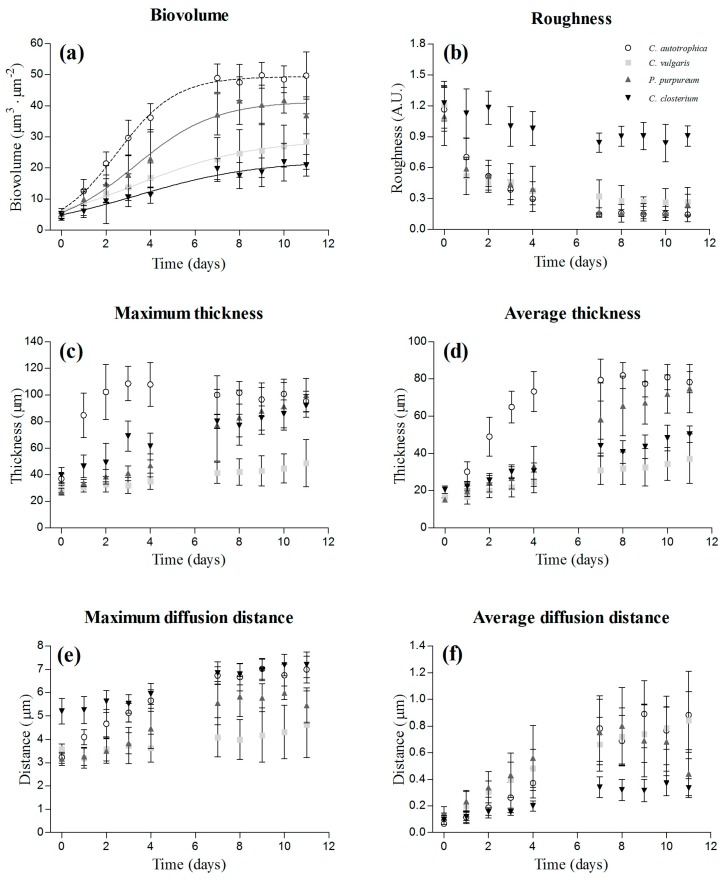
Dynamics of the structural parameters obtained from the z-stacks acquired at the confocal laser scanning microscopy (CLSM): biovolume (**a**), roughness (**b**), maximum thickness (**c**), average thickness (**d**), maximum diffusion distance (**e**), and average diffusion distance (**f**). The results are reported as the mean and standard deviation of 12 independent biological replicates. The fitting of the logistic model is also presented for the biovolume.

Examples of 3D biofilm reconstructions are reported in Figure 1a. The biovolume of the cells within the biofilm increased over time, and after seven days, the plateau was reached for all the species (Figure 2a). *C. autotrophica* and *P. purpureum* exhibited the highest biovolume, whereas *C. vulgaris* and *C. closterium* presented almost 50% lower biovolume. *C. autotrophica* also exhibited the highest growth rate, whereas no significant difference was found among the other microalgae (Table 1 and Figure 2a; *p* > 0.05).

Roughness presented an opposite trend to biovolume and decreased (from 1.2 to 0.2 a.u.) over time in *C. autotrophica*, *C. vulgaris*, and *P. purpureum*. Instead, the roughness coefficient of *C. closterium* remained stable at values around one (Figure 2b).

Over time, the increase in biomass resulted in a thickening of the biofilms (Figure 2c,d). *C. autotrophica* developed the thickest biofilms (~100 μm), and its maximum thickness remained stable over time. *P. purpureum* and *C. closterium* presented a similar increase of the maximum thickness reaching values such as those of *C. autotrophica*. *C. vulgaris* presented thinner biofilms with a maximum thickness of around 30–40 μm. The average thickness of *C. autotrophica* increased rapidly during the first four days, and then leveled off around 80 μm at day 7. *P. purpureum*, *C. closterium*, and *C. vulgaris* exhibited a more linear increase of the average thickness. *P. purpureum* reached a similar thickness to that of *C. autotrophica*, whereas *C. closterium* and *C. vulgaris* at the end presented 40–50% lower thicknesses with respect to *C. autotrophica*.

The maximum diffusion distance increased over time in all the species except for *C. vulgaris* (Figure 2e). At the end of the assay, *C. autotrophica* and *C. closterium* presented similar values and reached the highest values among all species, followed by *P. purpureum* and *C. vulgaris*. The average diffusion distance followed a similar pattern to biovolume: it increased rapidly during the first four days, and then leveled off from day 7 (Figure 2f). *C. autotrophica*, *C. vulgaris*, and *P. purpureum* presented comparable average diffusion distances (0.6–0.9 μm), whereas *C. closterium* exhibited the lowest (0.1–0.3 μm; *p* < 0.05).

### 3.2. Matrix Characterization: Lectins and Dextran Volumes

In order to characterize the matrix of the biofilms, the volume of the binding lectins and dextran was quantified. The volume of lectins reflected the fraction of glycoconjugates (EPS) in the matrix, and dextran was used to quantify the voids, water channels, and non-specifically the whole matrix (Figure 1b).

**Figure 3:**
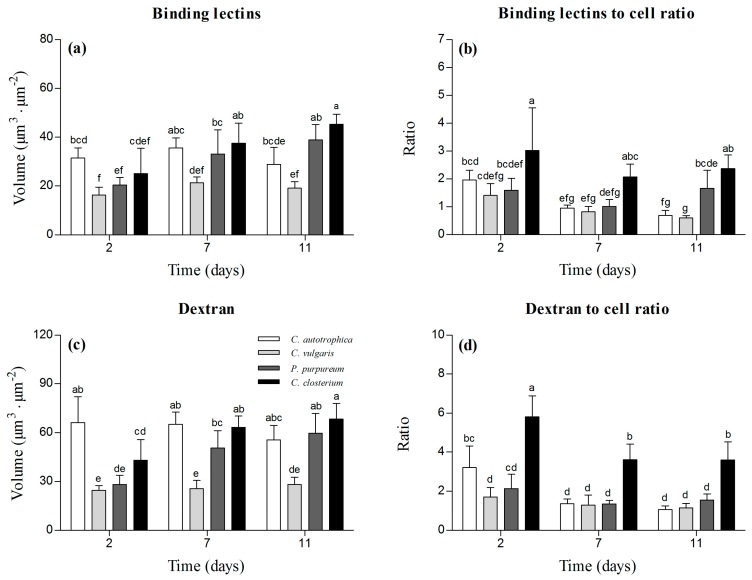
Structural parameters characterizing the biofilm matrix at days 2, 7, and 11. Volume of binding lectins ((**a**); specifically binding to glycoconjugates), lectin-to-cell ratio (**b**), volume of dextran ((**c**); used to stain the matrix non-specifically), and dextran-to-cell ratio (**d**). The results are reported as the mean and standard deviation of at least four independent biological replicates. Bars with different letters represent statistically different means (*p* < 0.05), as determined by pair-wise comparisons after two-way ANOVA.

*C. vulgaris* presented the lowest amount of EPS (Figure 3a), whereas it increased over time in *P. purpureum* and *C. closterium* biofilms, in which the volume of EPS doubled from day 2 to day 7. No temporal change was observed for *C. autotrophica* and *C. vulgaris* (*p* > 0.05). *C. closterium* presented the highest lectin-to-cell ratio (Figure 3b). Over time, *C. autotrophica* exhibited a decrease over time in the lectin-to-cell ratio (*p* < 0.05), whereas in the other species, the ratio remained stable.

At day 2, the volume of dextran was the highest in *C. autotrophica* biofilms (~65 μm^3^·μm^−2^). *P. purpureum* and *C. closterium* presented 50% and 30% lower volumes of dextran in their matrix, but at days 7 and 11, they approached the values of *C. autotrophica* (Figure 3c). *C. vulgaris* always presented the lowest volume of dextran in the matrix (~30 μm^3^·μm^−2^). In *C. autotrophica* and *C. vulgaris*, no significant change was observed over time (Figure 3c). Similarly to lectins, the dextran-to-cell ratio was always the highest in *C. closterium*, and it decreased from day 2 to 7 and then leveled off. A similar ratio was found for *C. autotrophica C. vulgaris*, and *P. purpureum*. *C. autotrophica* presented a decreasing trend from day 2 to day 7, similar to *C. closterium*. *C. vulgaris* and *P. purpureum* biofilms did not show any significant change over time in the dextran-to-cell ratio (Figure 3d).

### 3.3. Areal Coverage over Depth: Cells vs. Matrix Vertical Profiles

**Figure 4:**
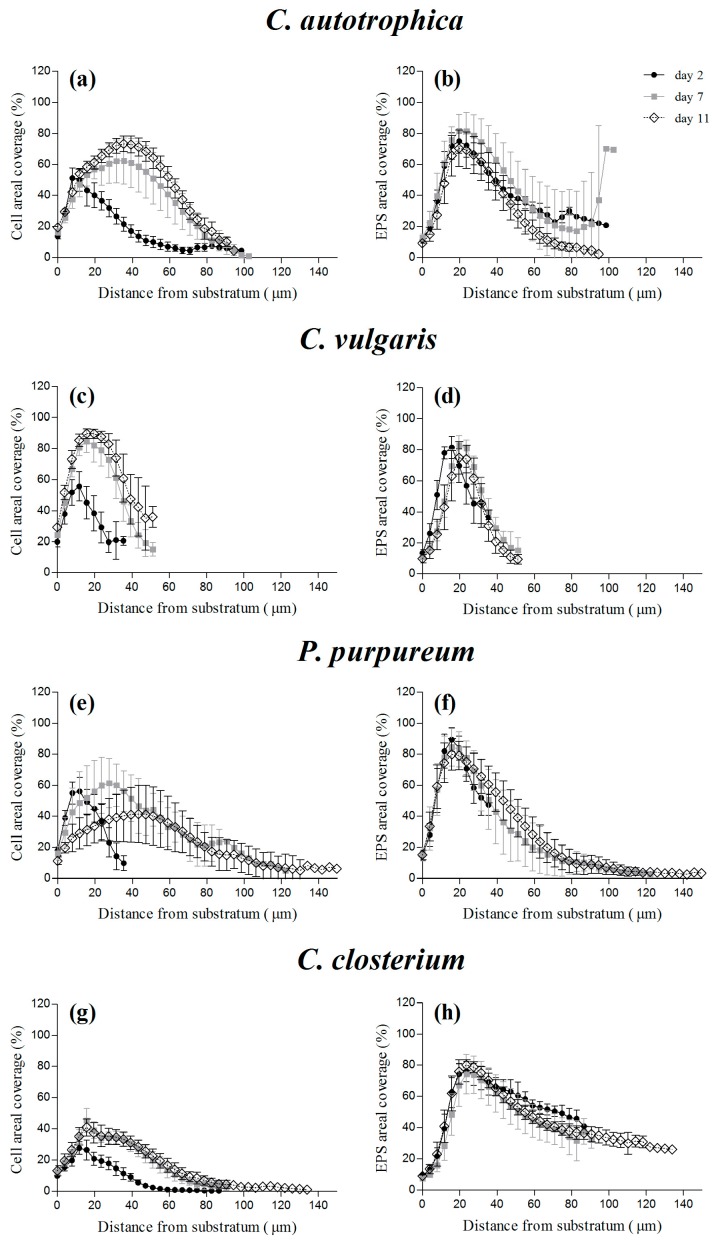
Vertical profiles of cells (**a**,**c**,**e**,**g**) and extracellular polymeric substances (EPS) (**b**,**d**,**f**,**h**) coverage of four different monospecific microalgae biofilms after 2, 7, and 11 days of maturation. The vertical profiles are reported as the percentage of coverage of cells or of EPS obtained from the z-stacks acquired at the confocal laser scanning microscopy (CLSM). The vertical profiles are reported as the mean and standard deviation of at least four independent biological replicates.

Regardless of the species, the maximum of cell density was reached at day 2, at a distance from the substratum of 10 μm, where the cells were already covering almost 50% of the area, except for *C. closterium*, which only covered 30% of the area (Figure 4g). On the other hand, during maturation, the maximal percentage of cell coverage moved at a greater distance (30–40 μm) above the substratum, and the depth at which it was reached and the percentage of coverage were species-specific (ranging from 40% to 90% of the covered area; Figure 4). Dextran and lectins presented comparable distributions over depth (Figure 4b,d,f,h and Figure S1). Interestingly, although the cells’ profile over depth changed as a function of time, the highest density of EPS and voids seemed to be positioned close to the substratum (20–30 μm) at all time points and for all the species.

In addition, the depth of the maximum cell density presented a positive correlation with the average biofilm thickness (R^2^ = 0.78, *p* = 0.0001). On the other hand, for the EPS, the regression slope was not significantly different from zero (Figure S2; R^2^ = 0.005, *p* = 0.81).

### 3.4. Biofilm Biochemical Characterization by ATR-FTIR Spectroscopy and Correlation Analysis with Structural Data

The average FTIR spectra of the biofilms at the different sampling times (days 2, 7, and 11) are reported in Figure S3. *C. autotrophica* and *P. purpureum* were the species that presented the greatest macromolecular changes over time.

**Figure 5:**
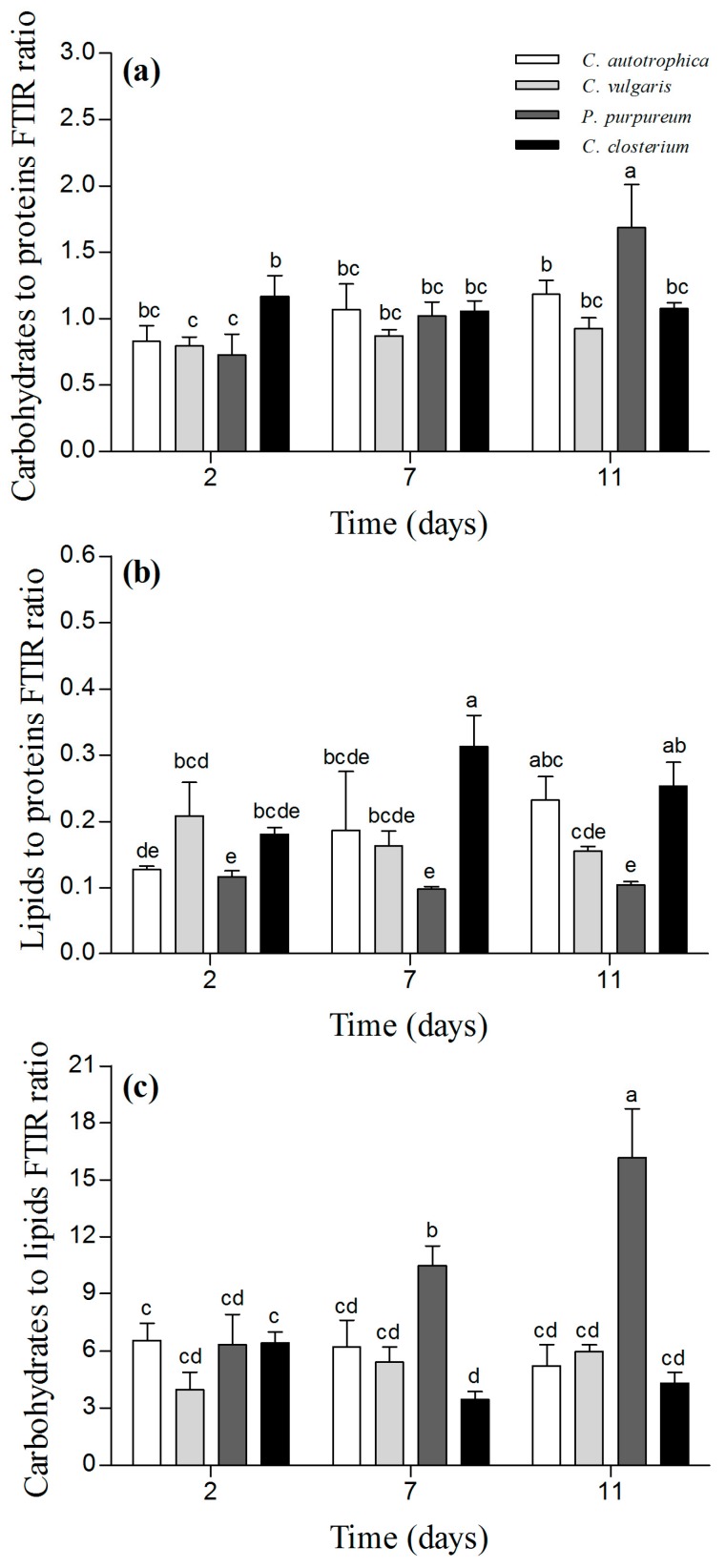
Macromolecular composition of the biofilms after 2, 7, and 11 days of maturation. Panel (**a**) depicts the carbohydrate-to-protein ratio, panel (**b**) depicts the lipid-to-protein ratio, and panel (**c**) represents the carbohydrate-to-lipid ratio. The results are reported as the mean and standard deviation of at least four independent biological replicates. Bars with different letters represent statistically different means (*p* < 0.05) as determined by pair-wise comparisons after the two-way ANOVA.

Similar carbohydrate-to-protein ratios were observed for all the samples, and the ratio did not change in *C. vulgaris*, *C. closterium*, and *C. autotrophica* (Figure 5a; *p* > 0.05). In contrast, for *P. purpureum,* the carbohydrate-to-proteins ratio was 50% higher at day 11 than that at day 2 (Figure 5a; *p* < 0.05). *P. purpureum* and *C. vulgaris* biofilms did not exhibit any change in the lipid-to-protein ratio (Figure 5b; *p* > 0.05). For *C. autotrophica* biofilms, the lipid-to-protein ratio increased by 50% at day 11 compared to day 2 (Figure 5b; *p* < 0.05), and for *C. closterium*, it increased by 30% at day 7 compared to day 2.

In *C. autotrophica* and *C. vulgaris* biofilms, the carbohydrate-to-lipid ratio did not change over time (*p* > 0.05). For *P. purpureum*, it tripled between day 2 and day 11. In *C. closterium*, the ratio was lower at day 7 compared to day 2 (almost 50%), but no difference was found between day 11 and day 2 (Figure 5c).

A correlation analysis was carried out to identify the possible correlations among the macromolecular ratios and the matrix of structural data (Figure S4). In *P. purpureum*, the EPS and dextran volumes were positively correlated to the carbohydrate-to-protein ratio and the carbohydrate-to-lipids ratio, whereas they were negatively correlated to the lipid-to-protein ratio. In all the other species, only minor correlations were found between matrix components and the macromolecular pools (Figure S4). In contrast, for these species, the macromolecular ratios were correlated to the structural parameters obtained from the cells’ autofluorescence such as thickness, biovolume, diffusion distance, and roughness (Figure S4).

## 4. Discussion

Our results show that the architecture of microalgae biofilms is species-dependent. However, the structure evolution over time seemed to follow some general common rules that resemble those described for bacteria and fungi [11,40,41,42].

During the first stages of substrate colonization, the biofilms were irregular in their surfaces (i.e., high roughness), thin, and for some of the species, the volume of EPS was higher than that of the cells (Figure 2 and Figure 3). As the biofilms matured, the biovolume increased, reflecting active cell division, similarly to what was reported for phototrophic biofilms by Mueller et al. [8] and Kernan et al. [20]. Mueller et al. [8] reported a linear increase, whereas our growth curves and those from Kernan et al. [20] reached a plateau (Figure 2a). Mueller et al. [8] focused on a natural mixed community including bacteria and different species of microalgae. Therefore, the species succession over time with different physiological requirements may have led to continuous growth. In our case and for Kernan et al. [20], the biofilms were composed by a single species that may have experienced over time energy or nutrient limitations, leading to a slowdown of the growth. Since nutrients were replenished every two days, light was probably the limiting factor. For instance, Barranguet et al. [23] reported that light was attenuated up to 90% in mature phototrophic biofilms [23], whereas Schnurr et al. [43] reported that the transmitted light for a biofilm with a thickness of 100 μm (similar to the ones reported in this study) was only 12% of the incident light. This means that for a photon flux density of 100 μmol photons·m^−2^·s^−1^, the cells in the deeper layers of the biofilm would receive only 12 μmol photons·m^−2^·s^−1^, which is an intensity far below the light compensation point measured for example in *C. vulgaris* biofilm (30–60 μmol photons·m^−2^·s^−1^; [44]).

The roughness of the biofilms decreased over time along with the progressive filling of the initial voids by new daughter cells (Figure 2b). A similar behavior has been described for natural multi-specific river biofilms [45] and for bacteria biofilms [10,41]. Mueller et al. [8], on the other hand, reported a positive correlation between biovolume and roughness and a parallel increase of diatoms over time. This is in agreement with the biofilm of the diatom *C. closterium*, which exhibited only minor changes of roughness over time (Figure 2b). The progression from rough to smoother surfaces as a biofilm matures has been proposed to be dependent either on the cells’ metabolic rate or on the maximum internal transport rate of nutrients [46,47]. In the case of cells with low metabolic activity (and therefore high nutrient availability), the valley between the biofilm peaks grows and merges with adjacent peaks, decreasing the roughness of the biofilm. Cells with high metabolic rates might become nutrient-limited, and division will proceed only at the biofilm peaks (i.e., along the vertical nutrient gradients [46,47]), inducing the formation of finger-like structures. Based on these observations, the development of smoother biofilms along time, in *P. purpureum*, *C. vulgaris*, and *C. autotrophica*, as compared to the rougher structured biofilms in *C. closterium*, may reflect different cell metabolic rates. Further metabolic investigations, such as intracellular measurements or O_2_ evolution, will be necessary to validate these conclusions.

As the majority of studies on photosynthetic biofilms focused on natural mixed communities (i.e., formed by bacteria and microalgae), little is known about EPS dynamics in microalgae biofilms. Here, we report the ability of microalgae to set up a supporting matrix and the dynamics of EPS during biofilm development. Interestingly, whereas the dynamics of cells were similar among all the species (Figure 2), those of EPS were more species-specific (Figure 3a): the two green algae did not show any quantitative change of the EPS, whereas *P. purpureum* and *C. closterium* presented a greater volume of EPS over time. These different trends are in agreement with the great variety of dynamics reported elsewhere as a function of the community composition. The trends of EPS production in photosynthetic biofilms that are present in the literature are indeed very disparate [8,23,24]. Ratios of matrix components (either considering lectins or dextran) to cells were not significantly different over time for all species, which may indicate a stable physiological state of the cells within the biofilm. Interestingly, they were positively correlated to the biofilm roughness coefficient, indicating that irregular surfaces were associated with high EPS content and low cell biovolume. Similar ratios of EPS to cells have been reported for stream-mixed biofilms by Battin et al. [45], who proposed that high EPS content may be advantageous for the attachment of the cells and first colonization of the substratum.

During biofilm development, several processes such as cell growth, EPS excretion, or consumption [48], as well as the establishment of chemical and physical gradients, may be responsible for the vertical distribution of cells and matrix components [9]. The few literature data about z-profiles in photosynthetic biofilms indicate that the distribution of cells and EPS is very much dependent on the culture conditions and on the biofilm nature (bacteria, algae, or mixed communities); nevertheless, some patterns can be drawn [8,18,20,23]. The maximal coverage for photosynthetic organisms seems to occur within the first 40 μm from the substratum, whereas EPS seem to match the cells’ position in young biofilms, and to be mostly placed in layers above the cells in older biofilms. In our work, at day 2, the cell z-profile was consistent with the patterns described in the literature: the maximal areal coverage of the cells was positioned 20 μm from the substratum (Figure 4), and the greatest coverage of EPS typically co-localized with the cells in proximity to the substratum (~20 μm). This means that the matrix components were mostly interspersed between the cells [49]. Over time, the maximal areal coverage of the cells moved at greater distances from the substratum, as described in Cole et al. [18], but the location of the maximum EPS density remained stable over time (Figure 4). Therefore, it is likely that the production of EPS in deep layers may have been responsible for the upward growth of the biofilm by global advection [48,50]. This is also supported by the maximum cell coverage moving upward as a function of the biofilm thickness, whereas the EPS maximal coverage remained stable (Figure S2).

In bacteria, changes in biofilms structures have been reported to be strongly related to qualitative shifts in the macromolecular composition of the EPS, which in turn may alter biofilms’ functions and properties [14,15,17,51]. In order to evaluate the relationships among macromolecules and structures in microalgae biofilms, a correlation analysis using the CLSM and FTIR results was performed. Lipids and/or carbohydrates changed in concentration as the biofilms matured and their architecture became more complex (Figure 5 and Figure S3). The lack of correlation between the macromolecular changes and the EPS and dextran volumes (except for *P. purpureum*) might indicate a reallocation of carbon in the cells rather than changes in matrix components. *P. purpureum* behaved differently, and such changes were positively correlated to the EPS and dextran volumes (Figure S4), suggesting an increase in glycoconjugates in the matrix as the biofilm matured [52].

Concluding, the development, architecture, and macromolecular composition of monospecific microalgae biofilms seem to be strongly species-dependent. Therefore, the selection of a microalgae strain for further cultivation in biofilm-based systems is a crucial step for the whole process, as specific structural features may be more or less advantageous under a certain set of culture conditions. Future studies will be required to address how hydrodynamics and the fluctuating light conditions, which are characteristic of outdoor cultivation systems, influence the structures and composition of algal biofilms. The data recorded in this study may be used to feed and calibrate photosynthetic growth models to better understand the mechanisms behind biofilm development under different conditions [53]. An extension of these models for large-scale production can be used for optimal process design or to guide the process operations. Furthermore, microfluidic tools or larger flow cells are promising tools for completing the picture and eventually providing an overview of the advantages and pitfalls of using microalgae biofilm-based systems [22].

## Figures and Tables

**Table 1:** Growth parameters (growth rate and maximal biovolume) obtained by fitting the logistic regression to the biovolume vs. time curves. Data are reported as the mean and standard deviation of at least nine independent biological replicates. Different letters represent statistically different means (*p* < 0.05) as determined by pair-wise comparisons after one-way ANOVA.

Microalgae Species	μ (d^−1^)	Maximal Biovolume (μm^3^·μm^−2^)
*C. autotrophica*	0.72 ^a^ (0.24)	50.47 ^a^ (4.09)
*C. vulgaris*	0.45 ^b^ (0.14)	31.22 ^c^ (8.49)
*P. purpureum*	0.65 ^ab^ (0.20)	43.02 ^b^ (4.92)
*C. closterium*	0.43 ^b^ (0.13)	22.42 ^d^ (2.59)

## Supplementary Materials

The following are available online at https://www.mdpi.com/2076-2607/7/9/352/s1, Figure S1: Vertical profiles of cells and dextran, Figure S2: Dependency of the depth of maximal coverage of cells and glycoconjugates (EPS) as a function of the average biofilm thickness, Figure S3: ATR-FTIR spectra of microalgae biofilms, Figure S4: Correlation plot, Table S1: Instrumental settings of the confocal microscope, Table S2: Biofilm structural parameters calculated using COMSTAT 2.1.
