# Supplementary material for: The Architecture of Monospecific Microalgae Biofilms
Source: Microorganisms. 2019 Sep 13;7(9):352. doi: 10.3390/microorganisms7090352 (PMC6780892; doi:10.3390/microorganisms7090352)
Supplement: Supplementary file 1 [file microorganisms-07-00352-s001.pdf]

microorganisms

MDPI

Supporting Information

Table S1. Instrumental settings of the confocal microscope used for the acquisition of the z-stacks of microalgae biofilms. The same instrumental settings were used for all the species at all time points in order to make the quantitative comparison of the images consistent. A.U. = Airy units.

CLSM parameters	Cells	Dextran	Lectins
Objective	20X	20X	20X
Zoom	0.5	0.5	0.5
Pinhole aperture	1 A.U.	1 A.U.	1 A.U.
Laser power	0.9%	7%	3%
Master Gain	630	670	670
Digital gain	1	1	1
Digital off-set	0	0	0
Scan speed	8	8	8
Scan time	1.56 sec	1.56 sec	1.56 sec
Pixel dwell	2.55 µsec	2.55 µsec	2.55 µsec
Averaging	0	0	0
Pixel size	x=1.25 µm; y=1.25 µm; z=3.94 µm	x=1.25 µm; y=1.25 µm; z=3.94 µm	x=1.25 µm; y=1.25 µm; z=3.94 µm

Table S2. Biofilm structural parameters calculated using COMSTAT 2.1 for the different biofilm components investigated in this study (i.e. cells, lectins and dextran). The threshold was automatically applied using the Otsu algorithm, pixels with an intensity below the threshold were considered as background whereas all other pixels were considered foreground.

Strtuctural parameters	Unit	Structural meaning	Cells	Dextran	Lectins
Biovolume	$\mu m^3 \cdot \mu m^{-2}$	Represent the total volume of signal voxels normalized for the area analyzed	*v*	*v*	*v*
Maximum thickness	µm	Distance from substratum of the most far pixel signal	*v*		
Average thickness	µm	The average distance from substratum of signal pixels	*v*		
Roughness	Arbitrary units	How much the thickness of the biofilms vary around the mean	*v*		
Maximum diffusion distance	µm	Longest distance between a voxel containing cell signal and a voxel not containing biomass	*v*		
Average diffusion distance	µm	average distance between a voxel containing cell signal and a voxel not containing biomass	*v*		
Percentage of area covered over-depth	%	Vertical profile of the percentage of area occupied by cells or matrix in each image of a z-stack	*v*	*v*	*v*

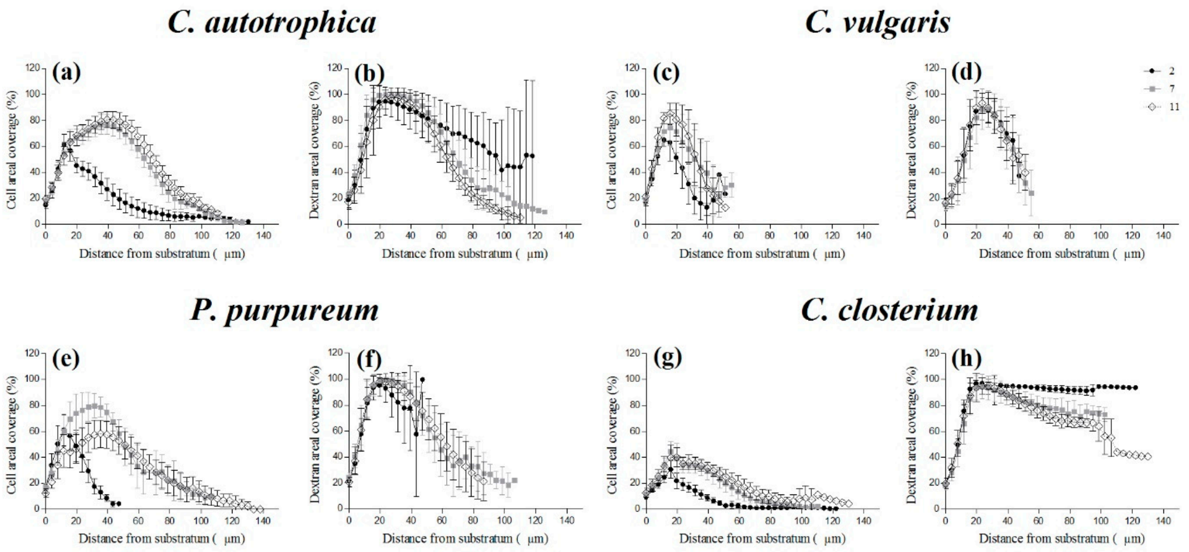

Figure S1. Vertical profiles of cells (a, c, e, g) and dextran (b, d, f, h) coverage of four different monospecific microalgae biofilms after 2, 7 and 11 days of maturation. The vertical profiles are reported as the percentage of coverage of cells or of dextran obtained from the z-stacks acquired at the CLSM. The vertical profiles are reported as the mean and standard deviation of at least four independent biological replicates. The values for each independent biological replicate were obtained by averaging the results of at least three z-stacks randomly acquired in a well.

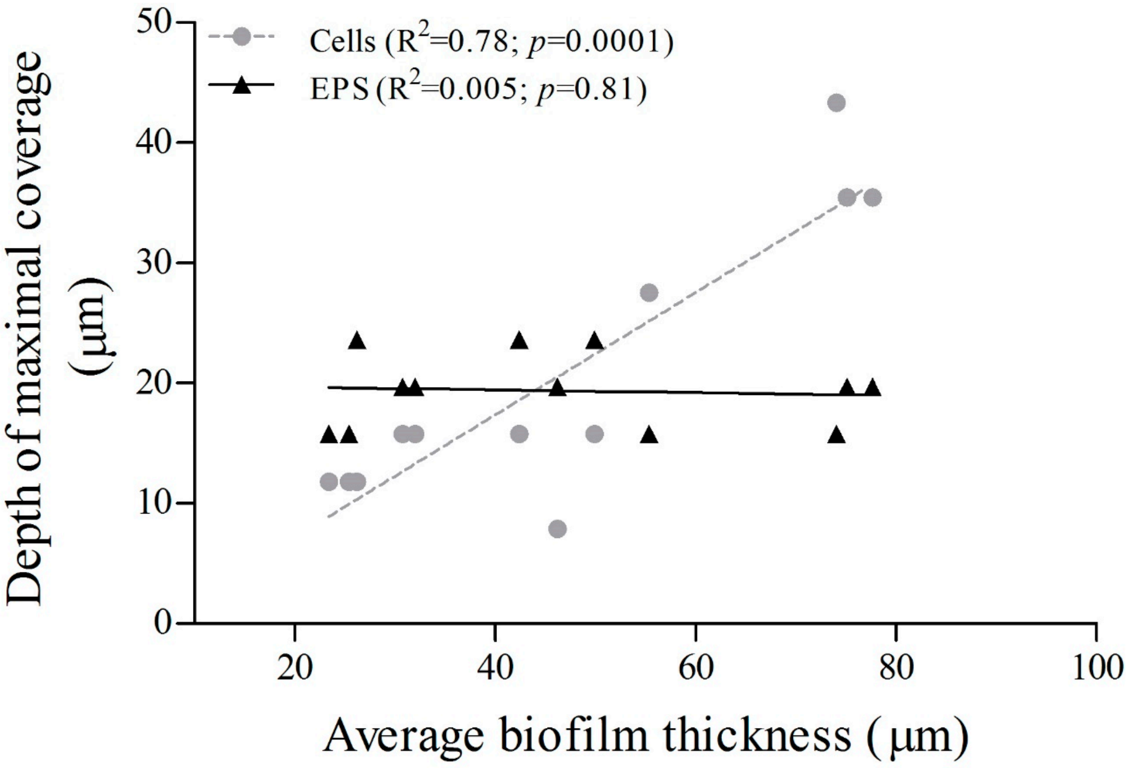

Figure S2. Dependency of the depth of maximal coverage of cells and glycoconjugates (EPS) as a function of the average biofilm thickness considering all the species together at day 2, 7 and 11. Linear regressions were performed to verify if the slopes were statistically different or not from zero. The R^2 and *p*-values of the linear regressions are also reported.

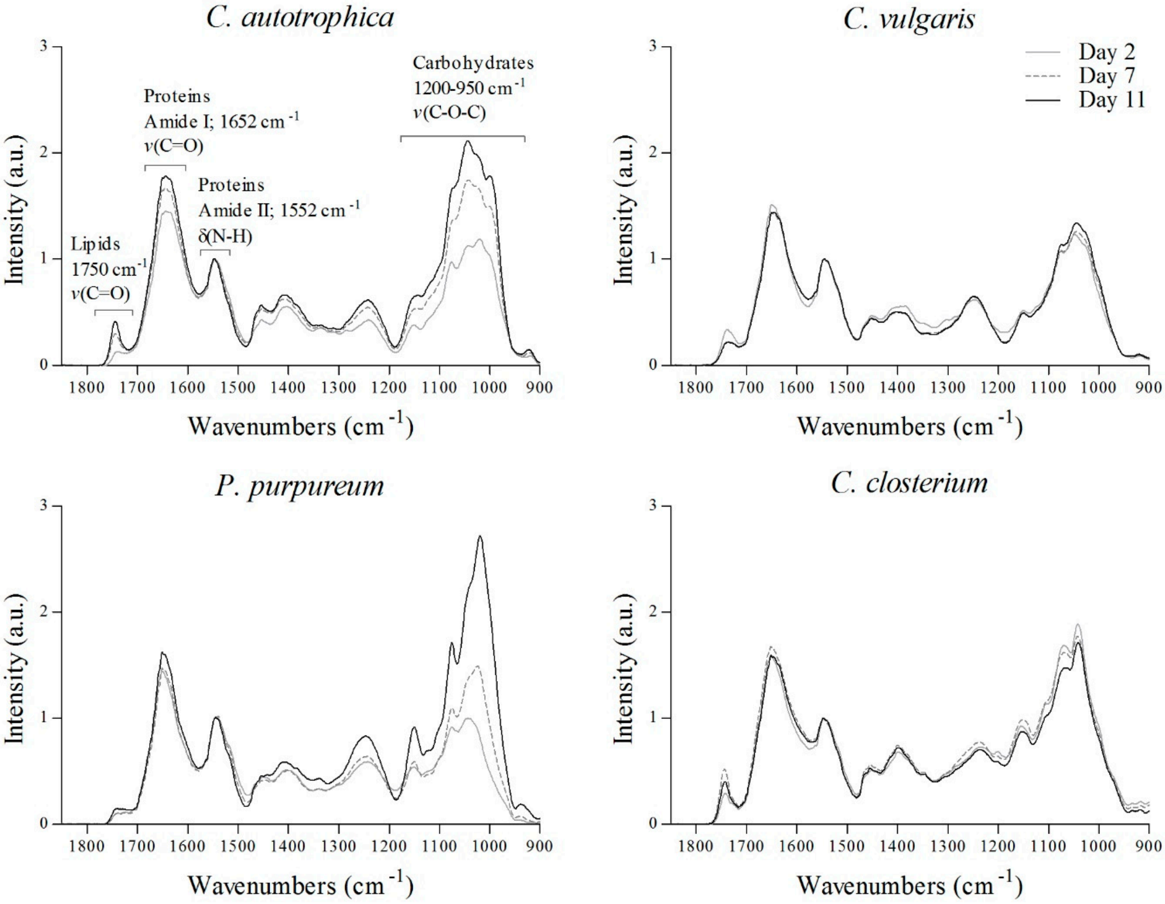

Figure S3. ATR-FTIR spectra of whole (cells and EPS) microalgae biofilms. The biofilms were harvested and measured at day 2, 7 and 11. The spectra are the average of at least four independent biological replicates and they were normalized to the Amide II band (~1552 cm^{-1}) in order to help for visual comparisons. The spectral regions corresponding to lipids, proteins and to carbohydrates are reported as an example in *C. autotrophica*. *ν* = stretching, *δ* = bending vibrational modes in infrared spectroscopy.

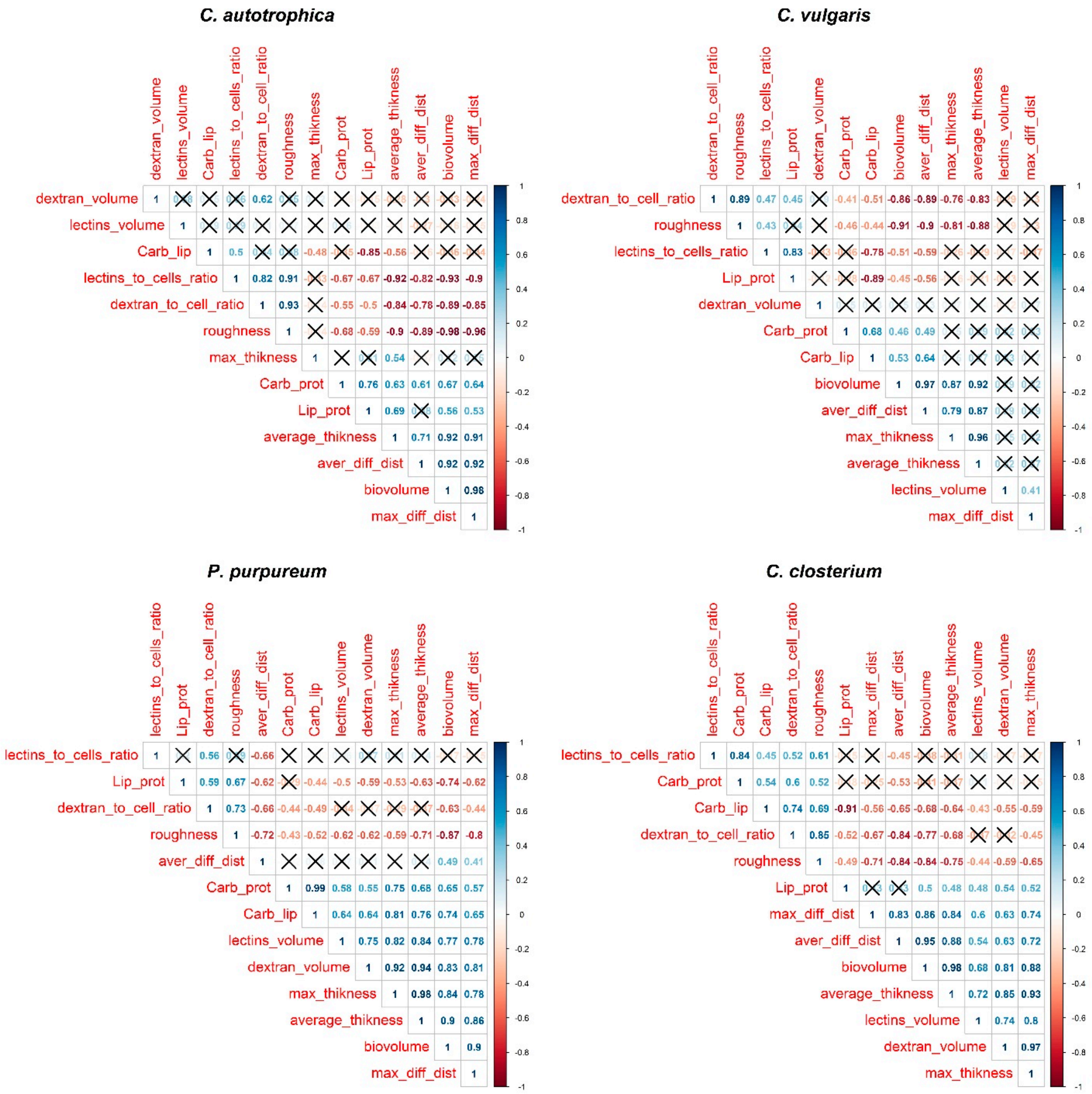

Figure S4. Correlation plot depicting the relationships among all the variables measured in this study for each of the microalgae. The method selected for the correlation was the Pearson's one, the blue color reflect a positive correlation and the red color reflect a negative correlation (see the correlogram at the right side of each graph). The "X" states that the correlation was found to be not statistically significant (p>0.05).
